# Electrical impedance tomography to monitor regional tidal ventilation at different pressure support levels

**DOI:** 10.1186/cc9566

**Published:** 2011-03-11

**Authors:** T Mauri, G Bellani, A Confalonieri, P Tagliabue, M Bombino, N Patroniti, G Foti, A Pesenti

**Affiliations:** 1 Universita degli Studi di Milano-Bicocca, Monza, Italy; 2 San Gerardo Hospital, Monza, Italy

## Introduction

Implementation of assisted mechanical ventilation in acute lung injury (ALI) patients may decrease ventilator-induced lung injury by redistribution of tidal ventilation towards dependent lung regions. Up to now, tidal ventilation regional distribution has been measured by expensive and complicated methods, not readily available at the bedside. Electrical impedance tomography (EIT) is a relatively new non-invasive bedside method to monitor tidal ventilation distribution, validated in preclinical studies. We verified the feasibility of using EIT to monitor tidal ventilation regional distribution in patients undergoing assisted ventilation and we describe the effect of different pressure support levels on regional ventilation.

## Methods

We enrolled 11 consecutive ALI patients admitted to our ICU, intubated and undergoing pressure support (PS) ventilation. We monitored the regional tidal ventilation distribution by means of a new EIT monitor (PulmoVista 500^®^; Dräger Medical GmbH, Lübeck, Germany), dividing the lung imaging field into four contiguous same-size regions of interest (ROI): ventral right (ROI 1) and left (ROI 2), dorsal right (ROI 3) and left (ROI 4). We randomly performed two steps of PS ventilation for 15 minutes, leaving the positive end-expiratory pressure (PEEP) and FiO_2 _unchanged: PS_low _(p0.1 ≥2 cmH_2_O) and PS_high _(p0.1 <2 cmH_2_O). At the end of each step, we recorded: ventilation parameters, arterial blood gas analysis and percentage of tidal ventilation distribution in the four ROIs. Analyses were performed by paired *t *test.

## Results

The ALI etiology was: trauma (18%), septic shock (18%), pneumonia (46%) and postoperative respiratory failure (18%). PS_low _was set at 3 ± 2 cmH_2_O and PS_high _at 12 ± 3 cmH_2_O. An increase in PS level determined a significant increase of tidal volume (7 ± 2 vs. 9 ± 3 ml/kg, *P *= 0.003) and peak inspiratory pressure (12 ± 4 vs. 18 ± 4 cmH_2_O, *P *= 0.0001). At PS_high_, proportional distribution of tidal ventilation significantly changed in all four ROIs (ROIs 1 to 4: 25 ± 9 vs. 33 ± 10%, *P *= 0.0003; 32 ± 13 vs. 37 ± 12%, *P *= 0.02; 20 ± 8 vs. 14 ± 8%, *P *= 0.0008; 23 ± 8 vs. 16 ± 5%, *P *= 0.005), moving from dorsal to ventral.

## Conclusions

EIT may be a useful tool to monitor lung regional ventilation at the bedside. PS levels that blunt patient effort may promote redistribution of tidal ventilation towards ventral lung regions.

